# Toll-like receptor (*TLR4*) Asp299Gly and Thr399Ile polymorphisms in relation to clinical falciparum malaria among Nigerian children: a multisite cross-sectional immunogenetic study in Lagos

**DOI:** 10.1186/s41021-015-0002-z

**Published:** 2015-06-16

**Authors:** Bamidele Abiodun Iwalokun, Afolabi Oluwadun, Senapon Olusola Iwalokun, Philip Agomo

**Affiliations:** Biochemistry and Nutrition Division, Nigerian Institute of Medical Research, 6 Edmund Crescent, Yaba. PMB. 2013, Lagos, Nigeria; Department of Medical Microbiology & Parasitology, Olabisi Onabanjo University, Sagamu, Ogun State Nigeria; Iru Primary Health Centre, Victoria Island, Lagos, Nigeria

## Abstract

**Introduction:**

This study determined the association of TLR4 Asp299Gly and Thr399Ile with uncomplicated and severe malaria among Nigerian children of similar ethnic background in Lagos. The association of these SNPs with high parasite density, malnutrition, hyperpyrexia and anaemia was also investigated.

**Methods:**

Genomic DNA of the study participants was screened for the genotypes of TLR4 Asp299Gly and Thr399Ile by PCR-RFLP. Anthropometric measurement was performed on the Pf infected children stratified into asymptomatic malaria (control), uncomplicated and severe malaria (case). Parasites were detected by light microscopy and Hardy Weinberg Equilibrium (HWE) of SNP genotypes was also determined.

**Results:**

A total of 279 children comprising 182 children (62.1 % male; mean ± SEM age, 57.3 ± 1.7 months) with clinical falciparum malaria and 97 children (55.7 % male; mean ± SEM age, 55.6 ± 2.5 years) with asymptomatic falciparum malaria were enrolled. All the genotypes of both TLR4 SNPs were found in the study population with their minor alleles: 299Gly and 399Ile, found to be 17.6 % and 14.7 % in severe malaria children. Unlike in asymptomatic population, the genotype distribution of TLR4 Asp299Gly SNP was not in HWE in the clinical malaria group but did not condition susceptibility. However, Asp299Gly and Thr399Ile polymorphisms were found to increase the risk of severe malaria 3-fold and 8-fold respectively (P < 0.05). They also increased the risk of severe anaemia, high parasite density and severe malnutrition 3.8 -5.3-fold, 3.3 – 4.4-fold and 4-fold respectively.

**Conclusions:**

Based on the above findings, we conclude that TLR4 Asp299Gly and Thr399Ile polymorphisms may modulate susceptibility to severe malaria among Nigerian children of Yoruba ethnic background.

## Introduction

Despite a reduction by 54 % in the African region since 2000, malaria due to *Plasmodium falciparum* still accounts for 18 % of child deaths [1, 2]*.* In 2012, 460,000 deaths out of the estimated 627,000 global malaria deaths were reported for children below 5 years with about 86 % of these deaths occurring in sub-Saharan Africa [2]. However, severe malaria occurs in less than 5 % of children below 5 years and this has been attributed to the host genetic factors. [2, 3]. Malaria is an inflammatory disease, which is initiated through recognition of parasite toxin such as glycophosphatidyl inositol (GPI) by innate immune cells such as monocytes, dendritic and macrophages for activation and elicitation of intracellular signal transduction pathway for the production of pro-inflammatory cytokines such tumor necrosis factor-alpha (TNF-α), interleukin-12 (IL-12) and interferon gamma (IFN-γ) [4, 5]. Timely and appropriate production of these cytokines in the early phase of infection and their down regulation by anti-inflammatory cytokines such as IL-10 in the later phase of infection has been shown to be crucial for parasitaemia control and avertion of host tissue damage and severe malaria [4, 5]. These events have been observed in infected African children who are non-susceptible compared to severe malaria susceptible children [6, 7]. Contrastingly, innate immunity dyregulation has been associated with risk factors and syndromes of severe malaria, including malnutrition, severe anaemia hyperparasitaemia or high parasite density [8, 9].

Toll-like receptor 4, a major pathogen recognition receptor (PRR) expressed on membrane surface of innate immune cells is genetically encoded by the *TLR4* gene (GeneID = 7099; 9q33.1), which spans a genomic region of ~13.3 kb with three exons (NCBI; http://www.ncbi.nlm.nih.gov/) [10] This PRR is a well-established receptor for the toxigenic GPI of *P. falciparum* and *Trypanosoma cruzi* as well as for lipopolysaccharide of gram negative bacteria and other pathogen associated molecular patterns (PAMPS) in gram positive bacteria, fungi and viruses [10–12]. Meanwhile, in vitro and animal model have shown the two most-studied non-synonymous single nucleotide polymorphisms (SNPs) of *TLR4*: an A to G transition (SNP ID = rs4986790), resulting in Aspartate-Glycine substitution at position 299 (*TLR4*Asp299Gly) and C to T transition (SNP ID = rs4986791), resulting in threonine-isoleucine substitution at position 399 (*TLR4*Thr399Ile), to cause altered GPI binding, 50 % reduction in *TLR4* expression on the membrane surface of innate immune cells, induce LPS hyporesponsiveness and excessive production of pro-inflammatory cytokines [11, 13, 14]. Subsequent case–control studies then established associations *TLR4* Asp299Gly or Thr399Ile polymorphism with death from septic shock and susceptibility to typhoid fever, tuberculosis, meningitis, chagas disease and respiratory syncytial virus infection in infected infants, children below 5 years and adults [11, 15]. However, findings from case–control studies in malaria endemic countries regarding association of these polymorphisms with susceptibility to clinical and severe malaria have been contradictory [16–23]. These discrepancies have been based on the differences geographical location and genetic background of human populations where these studies were conducted [16, 23]. Therefore, there is a need for more immunogenetic studies, regarding the role of *TLR4* polymorphisms in malaria pathogenesis, particularly in other countries with high malaria transmission.

Nigeria is currently among the top there high malaria burden countries in the world with an annual mortality of 300,000 deaths and where falciparum malaria accounts for 30 % of total under-five mortality every year [24]. Despite evidence from the HapMap project that the various genotypes of *TLR4* Asp299Gly and Thr399Ile SNPs are in circulation among the Yoruba tribe [25], the roles of these SNPs in influencing susceptibility to clinical and severe malaria remain unknown. It is on this basis that the present study was carried out to determine the frequency, distribution and association of mutant genotypes of *TLR4* Asp299Gly and Thr399Ile polymorphisms in a cohort of *P. falciparum* infected Nigerian children with susceptibility to clinical and severe malaria.

## Methods

### Study design and settings

This was a cross sectional study in which convenience sampling was used to enroll children during two separate malariometric surveys in Lagos between 2009 – 2011, the first survey was conducted in March – August, 2009 in Takway-Bay, Victoria Island, Lagos, while the second survey was conducted during the dry season in January 2011 in Ibeshe Community in Ikorodu. To further obtain *TLR4* SNPs data from severe malaria cases, children hospitalized at Massey Street Children Hospital, Lagos with laboratory and clinical evidence of severe falciparum malaria between September – October, 2011 were also enrolled into the study. Lagos is located within the Equatorial tropical region where malaria transmission mostly driven by female mosquitoes from the *Anopheles gambie* complex occurs throughout the year [26]. In Ibeshe, malaria prevalence rate of 14.2 % (95 % CI, 13.3 – 16 %) has been previously reported by Aina *et al.* [27], while in Takwa-Bay, children below 10 years account for 76.2 % of cases of uncomplicated malaria cases seen (Iwalokun *et al.*, unpublished). In this coastal settlement, malaria treatment practices were generally poor among caregivers despite their good knowledge of symptoms of uncomplicated malaria [28]. Massey Street Children Hospital is a foremost referral health facility of children in south West Nigeria where management of severe malaria on yearly basis is very common [29].

### Study population

The study population for this study comprised children (age < 13y) with *Plasmodium falciparum* positivity slide results. Asymptomatic malaria was defined as parasitaemia without fever in the previous 48 h or other related malaria symptoms or history of malaria in the preceding two months. Children in the uncomplicated malaria category were those having parasitaemia with fever (axillary temperature > 37.4 °C) other classical symptoms such as headache, chill and sweating plus mild-moderate anaemia (Hb < 11 g/dL - <9 g/dL), while severe malaria category were those with severe anaemia, fever plus one or more of other complications such as prostration, coma, jaundice, and respiratory distress [30]. At the time of enrollment, anthropometric measurements were performed with the children wearing light clothing and no shoes. Body length of children up to 23 months old was measured in recumbent position using a wooden horizontal stadiometer, while height of children aged 24 months and above was measured using a vertical stadiometer. These children were weighed using appropriate balance to the nearest 0.1 kg. The anthropometric measurements were then transformed into z scores with the aid of Epi-info 2000 software version 3.4 [31] and used for comparison with growth curve published by National Centre for Health statistics (NCHS) [32]. The z-score values for height for age (HAZ), weight for age (WAZ) and weight for height (WHZ) < −2 SD were defined as stunting, under weight and wasting based on the NCHS indices [32]. The field enrolled children with asymptomatic and uncomplicated malaria were treated with arthemether-Lumefantrine according to the national treatment guidelines, while those with severe malaria were referred to Massey Street Children’s Hospital for care with intramuscular loading dose of arthemether (3.2 mg/kg) given as a pre-referral treatment [33]. Children who did not have Yoruba ethnic background, with parasite negative glass slides and those whose caregivers declined consent were excluded from the study. This study was ancillary to the malariometric study that was approved by the institutional review board of the Nigerian Institute of Medical Research (NIMR-IRB), Lagos –Nigeria.

### Parasite detection

Parasite was detected and speciated by microscopic examination of thick (12 μL) and thin (3 μL) blood smear on grease-free labeled glass slides according to WHO guidelines. The glass slides were prepared in duplicates and read by two independent trained microscopists. A glass slide was considered parasite negative after examining 200 high power fields without seeing one parasite. Parasitaemia was measured by counting the number of parasites against 200–500 leukocytes using the thick blood film and expressed as number of parasites per 1 microlitre of whole blood, assuming 8000 leukocytes per 1 microlitre of blood.

### Blood haemoglobin measurement

A drop of finger pricked blood drawn into a microcuvette was used for the determination of haemoglobin (g/dL) using a Hemocue machine (Hemocue Hb 201). Anaemia was defines as Hb below 11 g/dL and in this study, Hb <7 g/dL was taken as severe malaria.

### Genomic DNA extraction

The salting out protocol of Miller *et al.* [34] was used for the extraction and purification of genomic DNA from peripheral blood samples of the study participants collected separately into labeled EDTA vials. DNA purification was done using the phenol-chloroform method.

### *TLR4* (Asp299Gly and Thr399Ile) genotyping

The alleles and genotypes of the two studied *TLR4* SNPs were determined by polymerase chain reaction-restriction fragment length polymorphism (PCR-RFLP) based method using the previously used primers sequences by Nyati *et al.* [35] (Table 1). All PCR amplification reactions were performed in a 20-uL volume PCR tubes containing 10X PCR buffer, 200 μM each of the dNTPs, 2.0 mM of MgCl_2_, 20 pmol of each primer, 1.25 U of Taq DNA polymerase (Promega, USA) and 100 ng of each genomic DNA as template. In a thermal cyler (Techne™ Thermal cycler TC-312, Fisher Scientific, UK), the PCR reaction was then subjected to denaturation at 94 °C for 5 min, followed by 35 cycles of denaturing at 94 °C for 30 s, annealing at 55 °C for 30 s, and extension at 72 °C for 30 s, and a final extension at 72 °C for 5 min and cooling to 4 °C. Template-free water was used as a negative control. After amplification, the purified 4-μL PCR products were subjected to restriction digestion by *Nco*I restriction endonuclease (Fermentas) for *TLR4* Asp299Gly and by *Hinf*I for *TLR4* Thr399Ile with 1 mL 10X enzyme buffer (Fermentas) overnight at 37 °C. The PCR products yielded 299 bp and 406 bp respectively, while the digested products yielded same DNA fragments for the wild alleles but decreased to 233 bp and 377 bp fragments for the 299Gly and 399Ile mutant alleles respectively, on 3 % agarose gel after electrophoresis (Table 1). Each restriction enzyme assay was duplicated for the confirmation of RFLP results.

**Table 1:** Nucleotide sequences of primers used and amplification conditions

Gene	Primer	Sequence	Amplification profile Temperature °C/time (sec)				Product size, bp	Restriction Enzyme	Cut Product size, bp
			D	A	E	C			
*TLR4*	299*TLR4*F	5′-GATTAGCATACTTAGACTACTACCTCCATG-3′	94 (30)	55 (30)	72 (30)	35	249	Nco1 (Fermentas)	W = 249
	299*TLR4*R	5-GATCAACTTCTGAAAAAGCATTCCCAC-3′							M = 223 + 26
	399*TLR4*F	5′-GGTTGCTGTTCTCAAAGTGATTTTGGGAGAA-3′	94 (30)	55 (30)	72 (30)	35	406	Hinf1 (Fermentas)	W = 406
	399*TLR4*R	5′-ACCTGAAGACTGGAGAGTGAGTTAAATGCT-3′							M = 377 + 29

### Statistical analysis

Data were double-entered into Microsoft Excel 2008 and Microsoft Access 2008 and validated before analysis using SPSS statistical software for windows version 15.0 (SPSS Inc, USA). Data were expressed as number and percentages (%), median and range, mean ± standard error of mean (SEM) and 95 % confidence intervals (95 % CI). High parasite density and hyperparasitaemia were defined as parasitaemia > 10,000/uL and > 250,000/uL respectively. Axillary body temperature > 40 °C was indicative of hyperpyrexia, while severe malnutrition was defined as WHZ or WAZ or HAZ < −3 relative to NCHS references. Disparity in mean values between asymptomatic control and clinical malaria case was measured by Student’s *t*-test, while allele and genotype frequency comparison was evaluated by chi-square (χ^2^) test using SPSS version 15.0 for windows. Association of *TLR4* Asp299Gly and Thr399Ile polymorphisms with clinical and severe malaria was measured by calculating odd ratio (OR) and their 95 % confidence intervals (95 % CI) using dominant inheritance model [23]. This was due to very low numbers of homozygous mutant genotypes of both TLR4 Asp299Gly and Thr399Ile polymorphisms. Therefore, they were grouped together with their respective heterozygotes as mutant genotypes for the calculation of odd ratios and their 95 % CI between clinical malaria cases and the asymptomatic control and between severe and uncomplicated malaria. Odd ratios and their 95 % CI without and with adjustment were also used to measure association of the TLR4 SNPs with the malaria disease phenotypes (*i.e.* anaemia, severe anaemia, malnutrition, severe malnutrition, high parasite density severe) measured as covariates. Adjustment was performed by including only cases of comparable ages between ASM and clinical malaria for anaemia, malnutrition (WHZ or WAZ or HAZ < −2) and gender or age for severe anaemia, severe malnutrition and high parasite density seen only in clinical malaria cases for the analysis of genotype distribution of the TLR4 SNPs. Hardy-Weinberg Equilibrium (HWE) was conducted by comparing of the observed frequencies of different genotypes of the two *TLR4* SNPs with their expected frequencies under HWE for each study group and outcomes with P > 0.05 was considered to be in HWE [36]. Genotypic deviation of HWE was measured by Pearson’s chi-square (χ2) statistical test. All statistical outcomes with P-value < 0.05 were considered to be significant.

## Results

A total of 279 children comprising 182 children (62.1 % male; mean ± SEM age, 57.3 ± 1.7 months) with clinical falciparum malaria and 97 children (55.7 % male; mean ± SEM age, 55.6 ± 2.5 months) with asymptomatic falciparum malaria were enrolled (Table 2).

**Table 2:** Socio-demographic, Parasitological and clinical characteristics of the *Plasmodium falciparum* infected children

Parameter	Study participants			*P*
	Total	Clinical malaria	Asymptomatic malaria	
	(n = 279)	(n = 182)	(n = 97)	
Age, median (Range), months	52 (8 – 110)	52 (8 – 110)	51 (10–105)	ND
Age, mean ± SEM, months	56.7 ± 1.4	57.3 ± 1.7	55.6 ± 2.5	0.59
Age group, n (%)				
6 – 24	14 (5)	8 (4.4)	6 (6.2)	0.54
25 – 59	152 (54.5)	101 (55.5)	51 (52.6)	0.69
60 and above	113 (40.5)	73 (40.1)	40 (41.2)	0.86
Gender				
Male, n (%)	167 (59.9)	113 (62.1)	54 (55.7)	0.29
Geometric mean parasite density (95 % CI), parasites/uL	5470 (2,170 – 7,770)	10,200 (5,300 – 15,100)	1,690 (1340 – 2040)	<0.001
Hyperparasitaemia				
(parasitaemia > 250,000/uL), n (%) High parasite density	2 (0.7)	2 (1.1)	0 (0)	ND
(parasitaemia >10,000/uL), n (%)	62 (22.2)	62 (33.2)	0 (0)	ND
Axillary body temperature, mean ± SEM, °C	37.1 ± 0.04	37.2 ± 0.05	36.8 ± 0.03	<0.0001
Hyperpyrexia (> 40 °C), n (%)	5 (1.8)	5 (2.7)	0 (0)	ND
Hb, mean ± SEM (range), g/dl	11.6 ± 0.1 (4.2 – 14.1)	11.3 ± 0.2 (4.2 – 13.4)	12.2 ± 0.1 (10.2 – 14.1)	<0.001
Anaemia (Hb < 11 g/dl), n (%)	49 (17.6)	37 (20.3)	12 (12.4)	ND
Severe anaemia, (Hb < 6 g/dl), n (%)	15 (5.4)	15 (8.2)	0 (0)	
Indices of malnutrition, n (%)				
WAZ (n = 224)			14 (16.9)	
< −2 SD	50 (22.3)	36 (25.5)	68 (81.9)	
−2 – to 2 SD	171 (76.3)	103 (73.0)	1 (1.2)	0.14
>2 SD	3 (1.4)	2 (1.5)		
HAZ (n = 239)			11 (13.3)	
< −2 SD	57 (23.8)	46 (29.5)	70 (84.3)	0.006
−2 – to 2 SD	176 (73.6)	106 (67.9)	2 (2.4)	
>2 SD	6 (2.6)	4 (2.6)	1 (1.2)	
WHZ (n = 215)				
< −2 SD	8 (3.7)	7 (5.3)	79 (96.3)	0.001
−2 – to 2 SD	204 (94.9)	125 (94.0)	2 (2.5)	
>2 SD	3 (1.4)	1 (0.7)		

ND = Not determined. Differences in malnutrition indices: weight for age WAZ), height for age (HAZ) and weight for height (WHZ) z scores between clinical and asymptomatic malarial children was evaluated with a 2 by 3 contingency table using chi-square (χ2) test. Disparity in mean values was evaluated using Student’s *t*-test. *P* < 0.05 was considered to be significant

The prevalence rates of underweight, stunting and wasting were found to be 22.3 %. 23.8 % and 3.7 % among the study participants. Among the children with clinical malaria, 2 (1.1 %), 5 (2.7 %), 15 (8.2 %) elicited hyperparasitaemia, hyperpyrexia and severe malaria. Between asymptomatic and clinical malarial children there was no statistical significance difference (P < 0.05) in terms of age, sex and being underweight. But variables such as stunting, wasting and axillary body temperature and anaemia occurred more significantly (P <0.05) in symptomatic children (Table 2).

All the genotypes of both *TLR4* SNPs were found in the study population (Tables 3 & 4). However, the difference in the proportions of genotypes of these SNPs between clinical malaria and asymptomatic control cases was not significant (P > 0.05) (Table 3). Further stratification of the clinical malaria cases showed that the frequencies of the minor alleles: 299Gly and 399Ile, of these *TLR4* SNPs were greater than 10 % (17.6 % and 14.7 % respectively) only in severe malaria children (Table 3 & 4).

**Table 3:** Genotype and minor allele frequencies and Hardy Weinberg equilibrium for the selected *TLR4* polymorphisms comparing children with asymptomatic and clinical malaria

*TLR4* SNP	Genotype/Minor allele	Study group			*OR (95 % CI)	P	HWE, χ2 (P)
		Total	Clinical malaria	ASM			
		(N = 279)	(n = 187)	(n = 92)			
Asp299Gly	Asp299Asp, % (Fraction)	88.5 (247/279)	87.7 (164/187)	90.2 (83/92)	1		Clinical malaria = 4.89 (<0.05)
	Asp299Gly, % (Fraction)	10.8 (28/279)	10.7 (20/187)	8.7 (8/92)			
	Gly299Gly, % (Fraction)	0.7 (4/279)	1.6 (3/187)	1.1 (1/92)	1.29 (0.57-2.92)	0.54	ASM = 0.11 (>0.05)
	MAF	6.5 (36/558)	7 (26/374)	5.4 (10/184)		0.49	
Thr399Ile	Thr399Thr, % (Fraction)	94.9 (265/279)	94.7 (177/187)	95.7 (88/92)	1		Clinical malaria = 1.35 (>0.05)
	Thr399Ile, % (Fraction)	4.3 (12/279)	4.3 (8/187)	4.3 (4/92)			
	Ile399Ile, % (Fraction)	0.8 (2/279)	1 (2/187)	0 (0/92)	1.24 (0.58-4.08	0.49	ASM = 0.21 (>0.05)
	MAF, % (Fraction)	2.9 (16/558)	3.2 (12/374)	2.2 (4/184)		0.50	

MAF = Minor allele frequency, OR = odd ratio, 95 % CI = 95 % confidence intervals, n = number of children screened per sub-group; N = total number of children screened. *OR was calculated after pooling heterozygous and homozygous mutant genotype frequencies; ASM = asymptomatic malaria; *P* < 0.05 was considered to be significant

**Table 4:** Genotype and minor allele frequencies and Hardy Weinberg equilibrium for the selected *TLR4* polymorphisms comparing children with uncomplicated and severe malaria

*TLR4* SNP	Genotype/Minor allele	Study group			OR (95 % CI)	P	HWE, χ2 (P)
		Total	SM	UM			
		(N = 187)	(n = 17)	(n = 170)			
Asp299Gly	Asp299Asp, % (Fraction)	87.7 (164/187)	70.6 (12/17)	89.4(152/170)	1		SM = 0.21 (>0.05)
	Asp299Gly, % (Fraction)	10.7 (20/187)	23.5 (4/17)	9.4 (16/170)			
	Gly299Gly, % (Fraction)	1.6 (3/187)	5.9 (1/17)	1.2 (2/170)	3.12 (1.11-11.12)	0.02	UM = 1.47 (>0.05)
	MAF	7 (26/374)	17.6(6/34)	5.9 (20/340)		0.01	
Thr399Ile	Thr399Thr, % (Fraction)	94.7 (177/187)	76.5 (13/17)	96.5 (164/170)	1		SM = 0.25 (>0.05)
	Thr399Ile, % (Fraction)	4.3 (8/187)	17.6 (3/17)	2.9 (5/170)			
	Ile399Ile, % (Fraction)	1.0 (2/187)	5.9 (1/17)	0.6 (1/170)	8.4 (2.1-33.6)	0.0005	UM = 0.59 (>0.05)
	MAF, % (Fraction)	3.2 (12/374)	14.7 (5/34)	2.1 (7/340)		0.00007	

MAF = Minor allele frequency, OR = odd ratio, 95 % CI = 95 % confidence intervals, n = number of children screened per sub-group; N = total number of children screened. *OR was calculated after pooling heterozygous and homozygous mutant genotype frequencies; SM = Severe malaria; UM = Uncomplicated malaria; *P* < 0.05 was considered to be significant

Unlike in asymptomatic population, the genotype distribution of *TLR4* Asp299Gly polymorphism was not in HWE in the clinical malaria group but did not condition susceptibility (Tables 3 & 4).

However, with stratification, Asp299Gly and Thr399Ile polymorphisms were found to increase the risk of severe malaria 3-fold and 8-fold respectively (P < 0.05) (Table 4). Further analysis revealed mutant genotypes of these SNPs to also increase the risk of severe anaemia, high parasite density and severe malnutrition 3.8 -5.3-fold, 3.3 – 4.4-fold and 4-fold respectively (Table 5).

**Table 5:** Associations between *TLR4* Asp299Gly and Thr399Ile polymorphisms and malaria outcomes, in children with clinical malaria

Association^a^	Prevalence % (fraction)	OR, 95 % CI, P	
		Unadjusted	Adjusted^b^
Anaemia			
*TLR4* Asp299Gly polymorphism			
Asp299Asp	28/164	1	1
Asp299Gly + Gly299Gly	9/23	3.12 (1.12 – 8.67) 0.01	2.87 (1.02 – 7.98), 0.02
*TLR4* Thr399Ile polymorphism			
Thr399Thr	31/177	1	1
Thr399Ile + Ile399Ile	6/10	7.06 (1.63 -32.1), 0.001	6.63 (1.53 – 30.15), 0.002
Severe Anaemia *TLR4* Asp299Gly polymorphism			
Asp299Asp	10/164	1	1
Asp299Gly + Gly299Gly	5/23	4.28 (1.12 – 15.8), 0.009	3.81 (1.07 – 13.1), 0.01
*TLR4* Thr399Ile polymorphism			
Thr399Thr	12/177	1	1
Thr399Ile + Ile399Ile	3 /10	5.89 (1.05 – 30.55), 0.0085	5.25 (0.93 – 27.3), 0.02
Malnutrition (WHZ or WAZ or HAZ < −2) *TLR4* Asp299Gly polymorphism			
Asp299Asp	34/164	1	1
Asp299Gly + Gly299Gly	8/23	2.04 (0.72 – 5.67), 0.13	1.93 (0.68 – 5.37), 0.16
*TLR4* Thr399Ile polymorphism			
Thr399Thr	39/177		1
Thr399Ile + Ile399Ile	3/10	1.1.52 (0.29 -6.95), 0.56	1.37 (0.27 – 6.31), 0.66
Severe malnutrition (WHZ; WAZ or HAZ < −3)			
*TLR4* Asp299Gly polymorphism			
Asp299Asp	10/97	1	1
Asp299Gly + Gly299Gly	4/12	3.87 (0.82 – 17.67), 0.037	3.70 (0.76 – 17.4), 0.05
*TLR4* Thr399Ile polymorphism			
Thr399Thr	12/100	1	1
Thr399Ile + Ile399Ile	2/9	2.11 (0.27 – 13.26), 0.38	1.79 (0.23 – 11.34), 0.43
High Parasite density			
*TLR4* Asp299Gly polymorphism		1	1
Asp299Asp	46/164	5.86 (2.09 – 16.96), 0.0007	3.54 (1.19 – 10.76), 0.009
Asp299Gly + Gly299Gly	16/23		
*TLR4* Thr399Ile polymorphism			
Thr399Thr	55/177	1	1
Thr399Ile + Ile399Ile	7/10	5.18 (1.15 – 26.38), 0.01	4.41 (0.97 – 22.54), 0.02
Fever *TLR4* Asp299Gly polymorphism			
Asp299Asp	48/164	1	1
Asp299Gly + Gly299Gly	10/23	1.86 (0.70 – 4.92), 0.16	0.88 (0.36 – 2.13), 0.76
*TLR4* Thr399Ile polymorphism			
Thr399Thr	52/177	1	1
Thr399Ile + Ile399Ile	6/10	3.61 (0.05 – 15.99), 0.04	4.27 (1.01 – 18.89), 0.02

OR = Odd ratio; 95 % CI = 95 % confidence interval; ^a^Dominant model of inheritance of the TLR4 genotypes was used for the analysis of their association with the disease phenotypes: anaemia, severe anaemia, malnutrition, severe malnutrition, high parasite density and fever. ^b^OR (95 % CI) adjustment was according to age and gender; *P* < 0.05 was taken as significant

## Discussion

The present study has examined the role of the two non-synonymous SNPs, rs4986790 (Asp299Gly) and rs4986791 (Thr399Ile), located at the third exon of the *TLR4* gene in the development of clinical and severe malaria among children with Yoruba ethnic background. This selection was based on evidence of existence of the two SNPs of *TLR4* among the Yoruba population (http://www.hapmap.org) [25]. For the first time in the setting of *P. falciparum* malaria, this study has also found all the genotypes of *TLR4* SNPs among children of Yoruba ethnicity. However, the difference in the numbers of mutant genotypes of both *TLR4* Asp299Gly and Thr399Ile SNPs between asymptomatic and clinical malarial children was observed to be non-significant (P < 0.05), translating to lack of association between carriage of these genetic variants and clinical malaria in the studied children. Our finding is similar to the report of Esposito *et al.* [37]. The workers also did not find significant association between the carriage of *TLR4* Asp299Gly SNP and malaria among Burundian children. The MAF of 6 % for 299Gly reported for the uninfected and malarial children by these workers is also similar to 5.4 – 7 % range found in this study. However, unlike in the Burundian children, we found MAF of 17.6 % and 10.4 % for *TLR4* Asp299Gly and Thr399Ile among our severe malaria sub-group with genotypes containing these alleles further found to increase the risk of severe malaria 3-fold and 8-fold respectively. Our findings indicate that the two minor alleles 299 Gly and 399Ile of TLR4 SNPs are not protective against severe malaria in the studied children. This is in agreement with the case–control studies conducted in Ghana [21]. In the Ghanian study, Mockhenhaupt *et al.* [21] reported also frequencies of 17.6 % and 24.1 % for these minor alleles in severe malarials children compared to 2.4 % and 6.2 % in healthy control. The workers also found these minor alleles to confer 1.5- and 2.6-fold increased risk of severe malaria respectively. In this study, we found the Asp299Gly heterozygote genotype in 8.7 %, 9.4 % and 23.5 % among the studied children with asymptomatic, UM and SM. Meanwhile, contrary to our findings and those of Mockenhaupt *et al.* [21], neither *TLR4* Asp299Gly nor Thr399Ile polymorphism was found to be associated with the risk of severe malaria anaemia and cerebral malaria in children from Uganda and Cameroun [17, 23]. This is in spite of the location of these countries in sub-Saharan Africa. This may not be unconnected with difference in ethnic background of these children and sample size coupled with temporal changes in malaria transmission, occurring in many malaria endemic African countries that might explain the different genotype and allele frequencies of *TLR4* Asp299Gly and Thr399Ile polymorphisms reported by these investigators. For instance, in the Cameroun study of 1,862 children, Apinjo *et al.* [23] reported MAF of 0.2 – 8.1 % for both *TLR4* polymorphisms in asymptomatic and clinical malaria (uncomplicated and severe malaria) children from Bantu, Foulbe and Semi-Bantu ethnic groups. The Ugandan study was conducted among 137 *P. falciparum* infected hospitalized children and only the frequency of Asp299Gly was above 10 % (*i.e.* 12.3 %) in the cerebral malaria group that also had a frequency of 1.5 % for the Thr399Ile genotype. This genotype was not found in children with uncomplicated malaria. In Brazil, where temporal changes from low to moderate *P. vivax* and *P. falciparum* malaria transmission also occur, contradictory findings, regarding the association of *TLR4* Asp299Gly polymorphisms with protection from clinical malaria have been reported [19, 20]. Within the Amazon region, da Silva *et al.* [19] found MAF of 5.8 % and 1.5 % for *TLR4*Asp299Gly polymorphism among 113 healthy and 535 clinical malaria participants with this polymorphism eliciting protection against clinical malaria, while Soares *et al.* [20] did not find protective effect of *TLR4*Asp299Gly among their 44 study participants in an earlier study.

Taken together, our findings and previous reports on *TLR4* Asp299Gly and Thr399Ile polymorphisms in malaria endemic countries strongly point to the relevance of other infectious diseases that also provide selection pressure on *TLR4*. They include tuberculosis, HIV, gram negative bacterial infection, candidiasis, meningitis and respiratory syncytial viral infection [5, 11]. Since the burden of these infectious diseases varies across the various malaria endemic countries, there is thus a high possibility of differences in their contributions to the evolution of mutant variants of *TLR4*.

Therefore, the implications of other TLR4 activated infectious diseases stated above highlights one of the limitations of the present study since the children were enrolled during malariometric surveys and were not clinically and diagnostically examined for candidemia and viral infections that may further influence the phenotypic disposition of TLR4 polymorphism and subsequently impact malaria susceptibility.

In this study and under dorminant model of inheritance, the significant association of the two TLR4 SNPs with clinical malaria syndromes such as fever, severe anaemia and high parasite density was found. This findings indicate that both TLR SNPs elicit functional effects that are related to the pathogenesis of falciparum malaria in the studied children. Similar phenotypic findings were also reported by Mochenhaupt *et al.* [22] in Ghanian pregnant women, the population of whom are also at high risk of clinical malaria globally. The workers found *TLR4*Asp299Gly polymorphism to increase maternal anemia 4.7-fold after adjustment by age. Here, we have found both *TLR4* Asp299Gly and Thr399Ile polymorphisms to cause a 3.8 -5.3-fold increased risk of severe anaemia and 3.5 – 4.4-fold increased risk of high parasite density, while Thr399Ile polymorphism alone raised the risk of fever 5-fold. In addition to Mockhenhaupt *et al.* [21], The phenotypic effects of TLR4 polymorphisms found in these study suggest that functional variations may exist between Asp299Gly and Thr399Ile SNPs of TLR4, regarding their systemic effects in Nigerian children with clinical malaria. Future studies that will look the combined effects of these SNPs through haplotype analysis will be very important. On the contrary, the functional effects of the two TLR4 SNPs observed in this study and in previous Ghanian study were absent in Camerounian children [23]. This disparity again confirm that malaria is a complex disease, involving multiple genetic factors that play different biological roles in disease manisfestation. Therefore, future haplotype-malaria association studies are needed to resolve inconsistency or heterogenous genotype-phenotype relationships in the setting of malaria in the African region.

However, of relevance is our finding that the mutant genotypes of *TLR4*Asp299Gly increased the risk of severe malnutrition 3.7-fold. However, this observation needs to be interpreted with caution because it was not been documented in previous immunogenetic studies of *TLR4* and malaria from other countries. Also because of the fact that malnutrition is a common health problem among African children, including Nigeria and many aetiologies have been reported [38, 39]. In fact, it has been reported that malnutrition accounts for 22 – 40 % of under –five mortality and malaria is one of the co-morbid factors [40, 41]. In the study, 3.7 %, of our study population as a whole elicited wasting, which indicates acute malnutrition 23.8 % were stunted, indicating chronic malnutrition and a state of long period of nutrient deprivation, while 22.3 % were underweight. Even in the setting of *P. falciparum* infection, these rates appear to be lower than the national prevalence rates 5.5 %, 35.7 % and 25.2 % and rates reported for children below 5 years in the northern parts of Nigeria. Another reason for this cautionary interpretation is the fact the present study is cross-sectional in design and is limited in determining the cause and effect relationship between malnutrition and malaria in the studied children. furthermore, the malnutrition investigated in this study exluded micronutrient deficiency such as zinc, iron and vitamin A deficiency, which have been shown to increase the risk of severe anaemia, wasting and dyregulated innate immunity in malarial children [42].

In conclusion, our findings suggest that *TLR4* Asp299Gly and Thr399Ile polymorphisms among Nigerian children of Yoruba ethnic background may modulate susceptibility to severe malaria and other disease outcomes such as severe anaemia and hyperpyrexia. To further validate the present findings, functional studies of TLR4 polymorphisms, haplotype analysis and investigation of other TLR4 activated infectious diseases in the setting of malaria in this category of Nigerian children are needed.
